# Pathologic Characteristics of Pregnancy-Related Meningiomas

**DOI:** 10.3390/cancers13153879

**Published:** 2021-08-01

**Authors:** Laura Giraldi, Emma Kofoed Lauridsen, Andrea Daniela Maier, Jørgen Vinsløv Hansen, Helle Broholm, Kåre Fugleholm, David Scheie, Tina Nørgaard Munch

**Affiliations:** 1 Department of Epidemiology Research, Statens Serum Institute, DK-2300 Copenhagen, Denmark; lvfg@ssi.dk (L.G.); jvha@ssi.dk (J.V.H.); 2 Department of Neurosurgery, Copenhagen University Hospital, DK-2100 Copenhagen, Denmark; emma.lauridsen@regionh.dk (E.K.L.); andrea.maier@regionh.dk (A.D.M.); kaare.fugleholm.buch@regionh.dk (K.F.); 3 Department of Pathology, Copenhagen University Hospital, DK-2100 Copenhagen, Denmark; helle.broholm@regionh.dk (H.B.); david.scheie@regionh.dk (D.S.); 4 Department of Clinical Medicine, University of Copenhagen, DK-2100 Copenhagen, Denmark

**Keywords:** meningioma, pregnancy-related meningioma, prolactin receptor, progesterone receptor, estrogen receptor, Ki-67

## Abstract

**Simple Summary:**

Meningiomas are the most common primary intracranial tumor in adults. Meningiomas are usually benign and slow growing. Treatment is surgical resection in the case of symptomatic growth. Dramatic growth can occur during pregnancy, complicating clinical management and entailing a risk to the well-being of the mother and fetus. Authors of a previous review paper raised the hypothesis that prolactin may be a key contributor to the sudden growth seen in pregnancy-related meningiomas. We set out to investigate the presence of prolactin receptors/prolactin, as well as other female hormones and histopathological characteristics of pregnancy-related meningiomas in Denmark, compared to meningiomas from female controls within the same age group. No differences in hormone receptor distribution were found between the groups and very few meningiomas expressed prolactin receptors, which contradicts the above-mentioned hypothesis. Interestingly, we observed above cut-point proliferative indices of the meningiomas for the entire study population of females in the reproductive age.

**Abstract:**

Meningiomas are the most common intracranial tumor. During pregnancy, explosive growth of a known meningioma occasionally occurs, but the underlying reasons remain unknown. Prolactin has been suggested as a possible key contributor to pregnancy-related meningioma growth. This study sets out to investigate prolactin and prolactin receptor status in 29 patients with pregnancy-related meningiomas in Denmark, from January 1972 to December 2016, as compared to 68 controls aged 20–45 years, also undergoing resection of a meningioma. Furthermore, we investigated potential differences in the progesterone and estrogen receptor statuses, WHO grade, Ki-67 labeling indices, and locations of the resected meningiomas between the cases and controls. Immunohistochemical analyses were performed, and histopathology and intracranial location were assessed with the investigator blinded for the case–control status. None of the samples stained positive for prolactin and very few samples stained positive for prolactin receptors, equally distributed among cases and controls. Estrogen and progesterone receptors generally followed the same distributional pattern between groups, whereas above cut-point Ki-67 labeling indices for both groups were observed. In conclusion, our results did not support the notion of prolactin as a key contributor to pregnancy-related meningioma growth. Rather, the similarities between the cases and controls suggest that meningiomas early in life may comprise a distinct biological entity.

## 1. Introduction

Meningioma (WHO Grades I–III) is the most common extra-axial, intracranial tumor. Meningiomas originate from arachnoid cap cells and may require surgical resection if the lesion is symptomatic and surgically accessible. The occurrence of meningioma is 5/100,000/year with an overweight observed in females [1]. The peak ratio of female to male incidence is 3.15 among those aged 35–44 years [2]. The age-specific incidence rate of meningioma is increasing throughout life, peaking at an incidence rate of 40/100,000 amongst women over the age of 85 years in the United States [3]. Only two known risk factors have been identified: neurofibromatosis type 2 and cranial ionizing radiation [1,4,5]. Female hormones have been suggested as a possible risk factor. However, several previous cohort studies found limited evidence of associations between female hormonal factors, such as parity, hormone replacement therapy, early menarche, and the occurrence of meningiomas [6,7,8,9,10,11,12,13,14,15,16,17,18]. It is well known that meningiomas express hormone receptors, but the composition of these varies. Up to 88% of meningiomas are progesterone receptor positive, 40% are estrogen receptor positive, and 39% are positive for androgen receptors [19].

The well-known phenomenon of explosive growth of an already known meningioma that occurs in some women during pregnancy remains puzzling [20,21,22]. A review points out that pregnancy-related meningiomas are more likely to be located near the pituitary gland than other meningiomas [23]. The authors speculate that meningiomas supplied by the anterior circulation may be exposed to prolactin. Prolactin passes through the fenestrated capillaries and the portal system, which is unique for the hypophyseal gland, and is possibly carried by the adjacent vessels along their natural paths, exposing nearby cells to high concentrations. Pregnancy-related accelerated growth is mainly observed during the second and third trimesters, which coincides temporally with increasing prolactin levels, thus supporting this notion [24,25,26]. Pregnancy-related meningiomas were more often of chordoid (6%) or clear-cell types (4%) [23], as compared to meningiomas in the background population, which were 0.5% [27] and 0.2% [28], respectively. Combined with the findings that 40–60% of meningiomas are prolactin receptor-positive [23], the authors raise the hypothesis that meningioma growth during pregnancy may be affected by prolactin.

The arguments underlying the hypothesis are (1) prolactin is secreted by the anterior pituitary gland [29], (2) the plasma concentration of prolactin during pregnancy reaches its peak at term [30], (3) prolactin accelerates the proliferation rate of meningioma-derived cells in vitro [31,32,33], (4) prolactin is both a circulating hormone and paracrine/autocrine factor participating in angiogenesis [34], which is important in the tumorigenesis process of, for example, meningiomas [35], and (5) prolactin has a pivotal role in osmoregulation in various cells and tissues [36,37]. It is speculated that this last feature could be responsible for the rapid changes in meningioma volume, which can be observed during and after pregnancy [23]. In summary, there are currently three underlying prolactin-related theories of drastic meningioma volume changes observed during and after pregnancy: angiogenesis, osmotic regulation and proliferative activity.

The Ki-67/MIB-1 monoclonal antibody is a marker of tumor proliferative activity correlating with histopathological malignancy grade [38] and is widely used in pathologic grading of meningiomas, affecting both diagnosis and prognosis [38]. In a literature study [38] from 2010, Ki-67 labeling indices were 4%, 8% and 17% for meningiomas WHO Grades I–III, respectively. A labeling index over 4% was associated with an increased recurrence rate [39]. In the aforementioned review in pregnancy-related meningiomas, a mean Ki-67 labeling index of 6.63% was found [23].

Studies investigating prolactin receptor status in meningiomas resected from pregnant or lactating women could not be found in the literature, possibly due to the extremely rare occurrence. The four neuropathology units in Denmark have stored tissue from all resected CNS tumors in Denmark since the 1970s, which provides the opportunity of investigating meningioma tissue removed from this patient group in a nation-wide case-control study. This will, by far, be the largest sample of pregnancy-related meningiomas as compared to previous studies.

The prolactin hypothesis described above proposes several distinct features of pregnancy-related meningiomas as compared to meningiomas from female controls aged 20–45 from the background population. The specific aims of this study were to investigate if pregnancy-related meningiomas were characterized by (1) specific histopathological features, (2) a higher expression of prolactin receptors and prolactin, (3) increased Ki-67 labeling index, and (4) the location of meningiomas in the skull-base area in close proximity to the pituitary gland. As secondary outcomes, the expression of estrogen and progesterone receptors were investigated.

## 2. Materials and Methods

### 2.1. Data Sources

The following registers were used:

The Civil Registration System (CRS) has existed since 1968 and is continuously updated with demographic information [40]. Every individual in Denmark is given a personal CPR-number, which allows interlacement of the different registers.

The National Patient Register (NPR) has registered all hospital admittances using the International Classification of Diseases (ICD-10) since 1977. Since 1995, emergency room visits, and outpatient contacts have been registered as well [41]. 

The Danish Cancer Registry (DCR) contains ICD-10 as well as histologic diagnoses of all types of cancer as defined in the WHO classification systems [42]. 

The Danish Pathology Register (DPR) was established in 1997 when all pathology departments in Denmark were legally obliged to report pathology data to the National Board of Health. Since 1999, DPR has been updated daily from the The National Danish Pathology Data Bank (DPDB) [43].

The National Danish Pathology Data Bank (DPDB) was established in 1999. Departments of pathology record all pathology data online to the DPDB, using guidelines published by the National Board of Health. Historical pathology data has been added to the database and, for a few departments, data from the early 1970s can be obtained [43].

### 2.2. Study Design

Retrospective observational study.

### 2.3. Study Population

The study population consisted of women aged 20–45 years, who underwent re-section of an intracranial meningioma during the period of 1 January 1972 to 31 December 2016, identified from the NPR, DCR, and DPR. Cases were women with pregnancy-related meningiomas who underwent operation during the study period. Pregnancy-related meningiomas were defined as meningiomas from women who underwent surgical resection no more than nine months before the date of giving birth and no later than 12 months after the date of giving birth. This time window was based on the normal duration of a pregnancy and the period of possible postpartum lactation. A randomly selected subset of women aged 20–45 years who underwent resection of a meningioma unrelated to pregnancy during the study period were used as controls. After cases and controls were identified in the registers, all available meningioma tissue samples were retrieved from the DPDB.

### 2.4. Exposure

Prolactin and pregnancy-related changes in the secretion of progesterone and estrogen. In terms of their pregnancy and the following period of possible lactation, the cases were exposed to much higher doses of prolactin than the control group [44].

### 2.5. Outcome

The primary outcome was positive immunohistochemical stains for the prolactin receptor and prolactin. Secondary outcomes were positive immunohistochemical stains for estrogen and progesterone, as well as Ki-67 labeling indices above cut-points for the respective WHO Grades I–III.

### 2.6. Pathology

Upon receival of the tissue samples and immunohistochemistry, the hematoxylin and eosin (HE) tissue stains were reviewed by a senior neuropathologist who evaluated which sample was most representative. Based on this, one sample per patient was chosen. The tissue samples were re-evaluated by two senior neuropathologists (HB, DS), according to the 2016 WHO classification and histopathological subtypes. The meningothelial and transitional subtypes were reported as a combined group since the two senior neuropathologists considered the distinguishment highly subjective. Furthermore, the distinction between meningothelial and transitional subtypes are of no clinical significance, with both subtypes belonging to WHO Grade I. The observers were blinded with regards to which patient and group (case/control) the pathology specimen belonged to.

The tissue samples were examined with immunohistochemical analyses for prolactin receptor (PRL-R), prolactin (PRL), estrogen receptor (ER), progesterone receptor (PR), and the Ki-67 labeling index.

### 2.7. Immunohistochemistry

The blocks were cut into 4 μm sections and fixated on glass. The PR staining was performed with BenchMark ULTRA incubation with a PGR antibody ((1E2), Roche 790-2223 Ventana Medical Systems, Basel, Switzerland) for 32 min. The positive control for PR was breast tissue. The ER staining was performed with BenchMark ULTRA, using an ER antibody ((SP1), Roche 790-2223 Ventana Medical Systems, Basel, Switzerland) and incubated for 32 min. The positive control for ER was breast tissue. The PRL-R staining was performed with BenchMark ULTRA incubation with a prolactin receptor antibody ((B6.2 + PRLR742) Abcam) for 30 min. The positive control for the PRL-R was a prolactin receptor positive prolactinoma. The PRL staining was performed with BenchMark ULTRA incubation with a prolactin antibody (poly, Roche 790-2223 Ventana Medical Systems, Basel, Switzerland) for 32 min. The positive control for PRL was the pituitary gland.

For the Ki-67 staining, tissue was incubated with mouse monoclonal anti-Ki-67 antibody (clone MIB-1 dilution 1:50, Agilent GA626 Dako Denmark A/S Glostrup). A standardized immunostaining procedure used an automated immunostainer (Dako Amnis, Agilent). The tissue samples were pre-heated (30 min at 97 °C) and a peroxidase blocking agent was added after primary antibody incubation (20 min.) The positive controls included human tonsil, colon or liver tissue.

### 2.8. Assessment of Tumor Labeling Index

The entire pathology specimen was systematically examined for PRL-R and PRL staining, using the ×40 lens. Tumor cells with a perinuclear stain were considered positive for PRL-R, even though stained cells were only found in dispersed solitary whorls.

ER expression was grouped in accordance with the American Society of Clinical Oncology/College of American Pathologists guidelines for breast cancer [45], where a ≥1% cut-off is considered positive. Therefore, specimens with ≥1% of unequivocally stained nuclei were placed in the “+” category. If over 50% of cells in a specimen were stained or multiple cells were heavily stained, these samples were placed in the category “++”. Samples without ER staining were placed in the “−“ category.

PR expression was divided into one of four categories by examining the full meningioma pathology specimen, using the ×40 lens: 0–25%, 25–50%, 50–75% and 75–100%.

Ki-67 was quantified by systematic examination of the entire pathology specimen and detection of stained hotspots. By viewing the hotspot using the ×40 lens, a hundred cells were counted, and the number of stained cells were expressed as the resulting percentage.

### 2.9. Statistical Analysis

For all levels of all categorical outcomes, the two-tailed Fisher’s exact test was used to test if the probability of being classified with a given level of positivity was the same for cases and controls. Within each level of WHO grade, a *t*-test allowing for unequal variances was used to test if means of the continuous outcome, the Ki-67 labeling index, was similar between cases and controls. A *p*-value of <0.05 was considered statistically significant. SAS 9.4 was used for data management and statistical analysis.

### 2.10. Ethics Statement

The study has been approved by the Danish Data Protection Agency and by the Regional Committees on Health Research Ethics in the Capital Region of Denmark (Journal No. H-18023804).

## 3. Results

### 3.1. Characteristics

**Figure 1:**
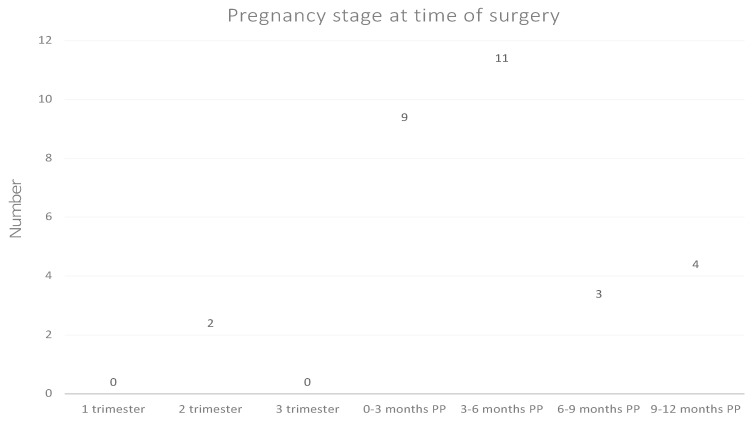
Temporal distribution of pregnancy-related meningioma surgeries in relation to pregnancy and postpartum period. PP = postpartum.

A total of 37 cases and 86 controls were identified from the National Patient Register and the Cancer Registry during the period 1 January 1972 to 31 December 2016. Tumor tissue could not be retrieved for eight cases and 18 controls, which resulted in a study population consisting of 29 women who underwent surgical resection of a meningioma during pregnancy or during the lactating period (up to 12 months after date of giving birth) and 68 females aged 20–45 who also underwent resection of a meningioma, but unrelated to pregnancy. Age at the time of surgery was not markedly different between cases and controls; the median age of the cases was 36 years, and the median age of the controls was 33 years. The majority of pregnancy-related meningiomas (76%) occurred in relation to first- or second-born child. Figure 1 presents the temporal distribution of pregnancy-related meningioma surgeries during pregnancy and the postpartum period.

### 3.2. Pathology 

#### 3.2.1. WHO Grades (I–III), Ki-67 Labeling Index and Histopathological Subtypes

Table 1 presents the distribution of pregnancy-related meningiomas and controls, according to the WHO grade, Ki-67 labeling index and histopathological subtype. A total of 89.7% of the pregnancy-related meningiomas were WHO Grade I and 10.3% were WHO Grade II (none were WHO Grade III). Of the controls, 83.8% were WHO Grade I, 13.2% were WHO Grade II and 2.9% were WHO Grade III.

Regarding the Ki-67 labeling index, the average values in the pregnancy-related meningiomas WHO Grade I was 6.3%, whereas the average for WHO grade II was 4.3% (three cases only). In the control group, the average Ki-67 values for WHO Grades I–III were 6.5%, 11.3% and 15.5% respectively.

Regarding the histopathological subtypes in the pregnancy-related meningiomas, 62.1% were meningothelial or transitional, which was similar to the controls (61.8%). Among the cases, 17.2% harbored fibrous meningiomas, which was found in 16.2% of controls. Microcystic meningiomas were found in 10.3% of cases but only 1.5% of controls. With regards to chordoid and clear cell meningiomas, which are of special interest, we found one of each among the cases, each corresponding to 3.4%.

#### 3.2.2. Hormone Receptor Status 

**Figure 2:**
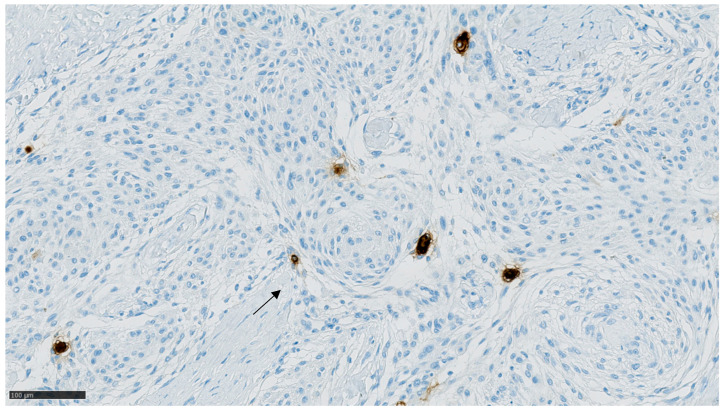
PRL-R staining in case at ×200 magnification. A few, dispersed tumor cells showed a strong, perinuclear stain.

Table 2 presents the expression of prolactin, prolactin receptors, estrogen receptors and progesterone receptors in cases and controls. Among pregnancy-related meningiomas, 6.9% expressed meningioma cells positive for prolactin receptors, compared to 2.9% of controls (*p* = 0.58). Positive meningioma cells were rare and widely dispersed, with a strong, perinuclear staining, as shown in Figure 2. No cases nor controls displayed positivity for PRL.

The estrogen receptor was overall positive in 27.6% of cases and 16.2% of controls, of which 27.6% of cases and 13.2% of controls were above the cut-point of ≥1% positive meningioma cells (*p* = 0.14). None of the pregnancy-related meningiomas were above the cut-point of >50%/many heavily stained cells, which applied only to very few of the controls (2.9%). 

**Figure 3:**
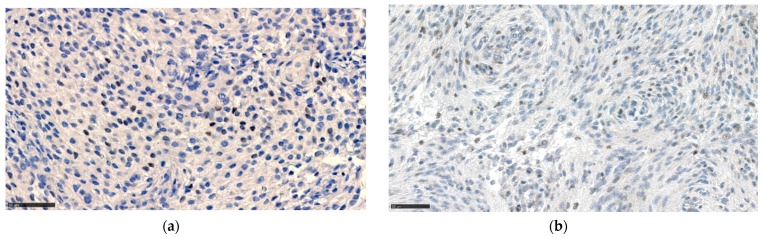
Estrogen receptor staining in meningioma tissue from (**a**) control and (**b**) case, both belonging to the “+” category at a ×400 magnification.

**Figure 4:**
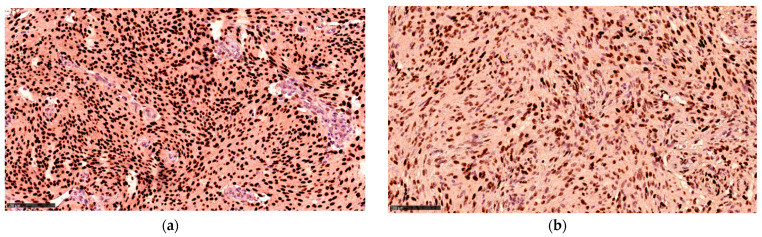
Progesterone receptor staining from (**a**) control and (**b**) case, both belonging to the 75–100% group at ×200 magnification.

The distribution of progesterone receptor expression in the cases was not significantly different from the controls. The staining patterns are presented in Figure 2, Figure 3 and Figure 4.

### 3.3. Meningioma Location

As previous studies suggest some association between intracranial location and the likelihood of hormonal impact on growth [23], we investigated the intracranial location for the subset of the study population in which this information was available. Thus, the meningioma locations by proximity to the pituitary gland, the location according to the major anatomical intracranial regions, are presented in Table 3.

No major differences were seen, however, of the pregnancy-related meningiomas; almost 60% of cases were located in the frontal/parasellar region, compared to 40% in controls (*p* = 0.044), whereas only 3.4% of the meningiomas from cases were located in the occipital region or posterior fossa, compared to 22% of the meningiomas from controls (*p* = 0.034).

## 4. Discussion

In this case–control study including the majority of patients with pregnancy-related meningiomas in Denmark 1972–2016, we investigated the presence of prolactin and the prolactin receptor status in the resected meningiomas, compared to meningiomas from female controls within the same age group. Furthermore, we investigated potential differences in the progesterone- and estrogen-receptor status, WHO grade, histopathological subtype, Ki-67 labeling indices and intracranial location of the resected meningiomas between the cases and controls. None of the samples stained positive for prolactin and very few meningioma cells stained positive for prolactin receptors, being equally distributed among pregnancy-related meningiomas and meningiomas from controls. Estrogen and progesterone receptors generally followed the same distributional pattern between groups, whereas above cut-point Ki-67 labeling indices for both cases and controls were observed. Thus, our results did not support the notion of prolactin being a key contributor to pregnancy-related meningioma growth. Rather, the similarities between the cases and controls may indicate that meningiomas displaying growth early in life constitute a distinct biological entity.

### 4.1. Pregnancy Stage and Replication History 

In this study, surgery was most often performed within 6 months postpartum (Figure 1). According to the previously mentioned review on pregnancy-related meningiomas, most of the meningiomas were diagnosed during the second and third trimesters of pregnancy [23]. As surgeries are preferentially carried out postpartum, it is possible that some of the meningiomas might have been diagnosed during pregnancy. Thus, the risks of surgery during pregnancy may very well account for the peak of surgeries in the first six months postpartum observed in this study. As we only had access to brain imaging studies for a minority of the patients, the exact time of diagnosis in our study population could not be investigated.

### 4.2. Pathology and Ki-67 Labeling Index

The distribution of benign and atypical meningiomas (WHO Grades I and II) were not significantly different between pregnancy-related meningiomas and the meningiomas from controls (Table 1). Notably, no malignant meningiomas were found among cases. Anaplastic (WHO Grade III) meningiomas constitute 1.0–2.8% of intracranial meningiomas [46], yet we found 15.5% among controls in the same age group. The higher occurrence of anaplastic meningiomas amongst this rather young population is noteworthy; however, it is based on a small number of patients.

Like Laviv et al., we found occurrence of the rare chordoid and clear-cell types of meningiomas (3.4% of each) among the pregnancy-related meningiomas. There may be a tendency toward overrepresentation in pregnancy-related meningiomas, considering the occurrences in the background population of 0.5% [27] and 0.2% [28], respectively. However, due to the small sample size, no firm conclusions about possible overrepresentation of chordoid and clear-cell meningiomas can be drawn. Furthermore, 10.3% of the pregnancy-related meningiomas were microcystic, compared to 1.5% among controls. Microcystic meningiomas share many similarities with clear-cell meningiomas [23].

The mean Ki-67 index in pregnancy-related meningiomas Grades I–II were 6.3% and 4.3%, respectively, which is in line with the mean Ki-67 index reported by Laviv et al. of 6.63% [23]. In our control group, mean Ki-67 labeling indices for meningiomas with WHO Grades I–III were 6.5%, 11.3% and 15.5%, respectively. These Ki-67 labeling indices are slightly higher than the reported general cut-off values of 4%, 8% and 17% for meningiomas of WHO Grades I, II, and III, respectively [38]. A Ki-67 labeling index over 4% has been described as a risk factor for recurrence of meningiomas [39]. This raises the hypothesis that not only pregnancy-related meningiomas, but perhaps also meningiomas in younger individuals in general, may have a higher proliferative activity than other meningiomas.

### 4.3. Hormone Receptor Status 

Only 6.9% and 1.5% of pregnancy-related meningiomas and controls expressed prolactin receptors (*p* = 0.58) in tumor cells. None of the samples stained positive for prolactin. These findings contradict the otherwise interesting and plausible hypothesis raised by Laviv et al., namely that prolactin drives meningioma growth during pregnancy, [23] as a greater amount of prolactin receptor positivity would have been expected in the pregnancy-related meningiomas. Furthermore, the few and widely dispersed PRL-R positive cells seem insufficient to account for meningioma volume changes by prolactin receptor-binding. Using more sensitive methods for detection of hormone receptor expression may be warranted in future studies.

These modest findings are also surprising in light of the previously reported prolactin receptor expression in up to 40–60% of meningiomas [32,33,47,48]. However, these studies utilized different methods for the detection of prolactin receptors, e.g., binding assays [32,33,47]. One study detected prolactin receptors in meningiomas by immunohistochemical methods, using a different antibody (clone PRL02; Neomarkers, Fremont, CA, USA) [48].

Thus, the lack of prolactin receptor expression and prolactin in meningioma cells makes prolactin an unlikely key contributor to the sudden growth seen in some pregnancy-related meningiomas, at least through the mechanism of prolactin–prolactin receptor binding. Laviv et al. also suggested that prolactin may contribute to mechanisms of angiomatous and edematous growth. However, no prolactin was discovered in the meningioma cells in this study. To our knowledge, there is no established association between prolactinoma and meningioma development or growth. In such cases, patients may be exposed to much higher concentrations of prolactin.

Our results regarding progesterone and estrogen receptor positivity are consistent with the reported expression of progesterone receptors in 88% of meningiomas from the general population and estrogen receptor expression in 40% [19]. No significant differences in estrogen and progesterone receptor distribution between cases and controls were found.

### 4.4. Meningioma Location 

A cornerstone in the hypothesis of prolactin-driven growth proposed by Laviv et al. is the location of pregnancy-related meningiomas. It was reported that meningiomas often have an anterior circulation blood supply. Information on the location of the meningiomas was available for 64% of this study population. Although not available for all, we do believe the proportion with available information is representative of the entire study population, as the lack of information is purely due to administrative causes. Similar to Laviv et al., who found that 68% of pregnancy-related meningiomas were found in the combined hypophyseal–sphenoid–olfactory region, we observed that meningiomas from cases were more prone to have similar locations (Table 3). One could ask the question of whether this indicates the influence of another pituitary hormonal factor. We did not investigate the presence of all the other pituitary hormone receptors, which could be the scope of a future study.

### 4.5. Limitations and Strengths of Study

An important limitation of this study is that the study involved old paraffin blocks; hence, we did not always know which fixation medium was used. This could potentially have influenced the immunohistochemical staining. 

This is the first nationwide investigation on pathological characteristics of the largest sample of pregnancy-related meningiomas to date. As pregnancy-related meningiomas are uncommon, it is highly valuable that departments of pathology have stored tissue samples, as this makes it possible to study a larger, unselected population.

## 5. Conclusions

In conclusion, the results of this study do not support the notion of prolactin being a key contributor to the sudden growth observed in some meningioma patients during pregnancy. Interestingly, both cases and controls displayed higher Ki-67 labeling indices than the cut-points of the respective WHO grades. Adding the fact that the cases and controls displayed fairly similar hormone receptor profiles and histopathological characteristics, altogether, they suggest that meningiomas from female patients in the reproductive age constitute a distinct biological entity in which a subset are prone to rapid growth. In general, neoplastic disease at a young age points toward a genetic predisposition. Future studies should be directed at elucidating the genetic and epigenetic characteristics of meningiomas among younger individuals in general.

## Figures and Tables

**Table 1:** WHO Grades (I–III), Ki-67 labeling index, and histopathological subtypes of pregnancy-related meningiomas and controls.

	Pregnancy-Related Meningiomas (N = 29) N (%)	Controls (N= 68) N (%)	*p*-Value **
WHO Grade			
I	26 (89.7)	57 (83.8)	0.54
II	3 (10.3)	9 (13.2)	1.00
III	0 (0)	2 (2.9)	1.00
Ki-67 labeling index (%) Mean value (SD)			
WHO Grade I	6.3 (6.0)	6.5 (5.0)	0.88
WHO Grade II	4.3 (2.9)	11.3 (7.4) *	0.043
WHO Grade III	NA	15.5 (13.4)	NA
Histopathological subtype			
Meningothelial/Transitional	18 (62.1)	42 (61.8)	1.00
Fibrous	5 (17.2)	11 (16.2)	1.00
Microcystic	3 (10.3)	1 (1.5)	0.079
Angiomatous	0 (0)	1 (1.5)	1.00
Metaplastic	0 (0)	1 (1.5)	1.00
Secretory	0 (0)	1 (1.5)	1.00
Chordoid	1 (3.4)	1 (1.5)	0.51
Clear cell	1 (3.4)	0 (0)	0.30
Atypical	1 (3.4)	8 (11.8)	0.27
Papillary	0 (0)	1 (1.5)	1.00
Rhabdoid	0 (0)	1 (1.5)	1.00

* One sample was excluded due to failed staining. ** For all levels of all categorical outcomes, the two-tailed Fisher’s exact test was used. Within each level of WHO grade, a *t*-test allowing for unequal variances was used to test if means of the continuous outcome, Ki-67 labeling index, was similar between cases and controls.

**Table 2:** Expression of prolactin, prolactin receptors, estrogen receptors and progesterone receptors in pregnancy-related meningiomas and controls.

Receptor Type		Pregnancy-Related Meningiomas (N = 29) N (%)	Controls (N= 68) N (%)	*p*-Value ^#^
Prolactin receptor		2 (6.9)	2 (2.9)	0.58
Prolactin		0 (0)	0 (0)	1.00
Estrogen *	−	21 (72.4)	57 (83.8)	0.26
	+	8 (27.6)	9 (13.2)	0.14
	++	0 (0)	2 (2.9)	1.00
Progesterone **	0–25%	4 (14.3)	14 (20.9)	0.57
	25–50%	4 (14.3)	6 (9.0)	0.47
	50–75%	3 (10.7)	10 (14.9)	0.75
	75–100%	17 (60.7)	37 (55.2)	0.66

* In the “−“ group, there was no ER expression. In the “+” group, a cut-off value of ≥1% of ER expression was used, and in the “++” group, either >50% of cells expressed ER receptors or many cells were considered heavily stained. ** In one case and one control, the progesterone staining was considered insufficient, and thus these were excluded from the results. ^#^ Estimated by Fisher’s exact test.

**Table 3:** The meningioma location by frontal/parasellar location, and according to the major anatomical intracranial regions.

Location by Proximity to the Pituitary Gland	Pregnancy-Related Meningiomas (N = 29) N (%)	Controls (N= 68) N (%)	*p*-Value ^#^
Frontal/parasellar region *	17 (58.6)	24 (35.3)	0.044
Other **	2 (6.9)	17 (25.0)	0.051
No information	10 (34.5)	27 (39.7)	0.66
**Location according to the major anatomical intracranial regions**			
A: Frontoparietal, olfactory groove, “fossa anterior” and frontal convexity	7 (24.1)	16 (23.5)	1.00
B: Sphenoid wing, suprasellar, temporofrontal and tuberculum sellae	10 (34.5)	10 (14.7)	0.052
C: Occipital and fossa posterior	1 (3.4)	15 (22.1)	0.034
No information	11 (37.9)	27 (39.7)	1.00

* Frontal/parasellar region: fossa anterior, frontal, frontoparietal, sphenoid wing, OGM, opticus, sella, temporofrontal, clinoideus anterior, orbita. ** Other: ventricles, occipital, CPV, fossa media, parietal, posterior, sinus transversus, temporal, trigeminus. ^#^ Estimated by Fisher’s exact test.

## Data Availability

The data presented in this study are available on request from the corresponding author. The data are not publicly available due to restrictions from the Danish Data Protection Agency.
